# Arbitrage Equilibrium, Invariance, and the Emergence of Spontaneous Order in the Dynamics of Bird-like Agents

**DOI:** 10.3390/e25071043

**Published:** 2023-07-11

**Authors:** Abhishek Sivaram, Venkat Venkatasubramanian

**Affiliations:** 1 Department of Chemical and Biochemical Engineering, Technical University of Denmark, 2800 Kgs. Lyngby, Denmark; abhisi@kt.dtu.dk; 2 Complex Resilient Intelligent Systems Laboratory, Department of Chemical Engineering, Columbia University, New York, NY 10027, USA

**Keywords:** self-organization, pattern formation, maximum utility, flocking, murmuration

## Abstract

The physics of active biological matter, such as bacterial colonies and bird flocks, exhibiting interesting self-organizing dynamical behavior has gained considerable importance in recent years. Current theoretical advances use techniques from hydrodynamics, kinetic theory, and non-equilibrium statistical physics. However, for biological agents, these approaches do not seem to recognize explicitly their critical feature: namely, the role of survival-driven *purpose* and the attendant pursuit of *maximum utility.* Here, we propose a game-theoretic framework, statistical teleodynamics, that demonstrates that the bird-like agents self-organize dynamically into flocks to approach a *stable arbitrage equilibrium*of equal effective utilities. This is essentially the *invisible hand*mechanism of Adam Smith’s in an ecological context. What we demonstrate is for ideal systems, similar to the ideal gas or Ising model in thermodynamics. The next steps would involve examining and learning how real swarms behave compared to their ideal versions. Our theory is not limited to just birds flocking but can be adapted for the self-organizing dynamics of other active matter systems.

## 1. Self-Organization in Active Matter: Background

Active matter describes systems composed of large numbers of self-actualizing agents that consume and dissipate energy resulting in interesting dynamical behavior [1,2,3,4,5,6,7,8,9]. Biological examples of such systems include self-organizing bio-polymers, bacteria, schools of fish, flocks of birds, and so on. This study is focused on birds flocking.

Flocking has been studied extensively from dynamical systems and statistical mechanics perspectives [10,11,12,13,14]. Such analyses have contributed substantially to our evolving understanding of interesting emergent properties such as phase segregation, flock stability, etc. However, these approaches typically do not seem to model explicitly the critical feature of active biological agents, namely, the role of *purpose* and its attendant *pursuit of maximum utility*. Being biological agents, birds are innately purposeful, driven by the goal to survive and thrive in challenging environments as Darwin explained. We believe any comprehensive theory of active biological matter needs to overtly account for this defining characteristic of these agents.

We address this need by using a game-theoretic framework, which we call *statistical teleodynamics* [15,16,17,18,19,20]. The name comes from the Greek word *telos*, which means goal. Just as the dynamical behavior of gas molecules is driven by thermal agitation (hence, *thermo*dynamics), the dynamics of purposeful agents is driven by the pursuit of their goals and, hence, *teleo*dynamics. Statistical teleodynamics may be considered as the natural generalization of statistical thermodynamics for *purpose-driven* agents in active matter. It is a synthesis of the central concepts and techniques of potential games theory with those of statistical mechanics toward a unified theory of emergent equilibrium phenomena in active and passive matter [20].

As noted, there is considerable literature on the Reynolds and Vicsek models of birds flocking. Our purpose here is not to reproduce the results of these models, although our approach, too, leads to similar flocking behavior. Our goal is to propose an alternative modeling framework that explicitly accounts for the goal-driven behavior of biologically active agents. Furthermore, our framework answers important questions that are not addressed by the Reynolds model: (i) Is there an equilibrium outcome of this dynamics? (ii) If yes, what is the nature of the equilibrium? (iii) What is the equilibrium configuration? and (iv) Is the equilibrium stable? In addition, our formulation also has the benefit of readily generalizing to other active matter systems in biology, ecology, sociology, and economics, as we describe in the discussion section.

We wish to emphasize that the spirit of our modeling is similar to that of the ideal gas or the Ising model in statistical thermodynamics. Just as real molecules are not point-like objects or devoid of intermolecular interactions, as assumed in the ideal gas model in statistical mechanics, we make similar simplifying assumptions in our bird model. These can be relaxed to make them more realistic in subsequent refinements as van der Waals did, for example, in thermodynamics. The ideal versions serve as useful starting and reference points to develop more comprehensive models of self-organization in active matter systems.

## 2. Statistical Teleodynamics of Flocking: A Game-Theoretic Formulation

In modeling the dynamics of flocking, there are two dominant approaches: (i) the bottom–up agent-based modeling perspective, as seen in the Reynolds’ Boids model [10] and the Vicsek model [11], and (ii) the top–down statistical mechanics formulation that starts with the specification of the Hamiltonian of the flock and then imposes the maximum entropy distribution on it [13,14].

The Reynolds and Vicsek models specify agent-level dynamical behavior (such as the three rules of cohesion, separation, and alignment for boids). However, they do not predict a final equilibrium state, as there is no notion of equilibrium. The final state is determined only computationally via agent-based simulations.

On the other hand, in the statistical mechanics formulation, it is not clear why maximum entropy, which is obviously relevant for passive matter systems, would be applicable for survival-driven birds. The typical statistical mechanics approach uses the superficial similarity between spins in magnetic systems (e.g., the Ising model) and the orientation of the birds to apply maximum entropy methods. The deeper question of why is this conceptually relevant for birds is not addressed. Most importantly, all these approaches do not seem to recognize explicitly that active agents such as birds act instinctively to improve their survival prospects.

We address these challenges using our statistical teleodynamics framework [15,16,17,18,19,20]. In this theory, the fundamental quantity is an agent’s *effective utility*, which is a measure of the net benefits pursued by the agent. Every agent behaves strategically to increase its effective utility by switching states and exploiting arbitrage opportunities. In our theory of flocking, we propose that birds are arbitrageurs that maneuver to increase their effective utilities dynamically in flight. The effective utility of a bird depends on its position, speed, and alignment with the rest of the members in its neighborhood.

Hence, we believe that the proper formulation of flocking (in general, any herding behavior) ought to start with a model of effective utility that an agent uses to make such decisions dynamically in flight. Seen from this perspective, we suggest that birds do not fly *randomly* (as statistical mechanics-based formulations implicitly assume) but maneuver *strategically* to improve their utilities. We exploit this insight to model the dynamical behavior of birds in flight by using the concepts and techniques from potential games.

In potential games, there exists a single scalar-valued global function called a potential (ϕ(x)) that has the necessary information about the payoffs or the utilities of the agents. The gradient of the potential is the utility, $h_{ik}$, of the ith agent in the kth state [15,21,22,23,24]. Therefore, we have

(1) $$h_{ik}\left(x\right)\equiv \partial \phi (x)/\partial x_{k}$$

where $x_{k}=N_{k}/N$ and x is the population vector, where $N_{k}$ is the number of agents in state *k*, and *N* is the total number of agents. A potential game reaches equilibrium, called Nash equilibrium (NE), when the potential ϕ(x) is maximized. Furthermore, this Nash equilibrium is unique if ϕ(x) is strictly concave [22]. At Nash equilibrium, all agents enjoy the same effective utility, i.e., $h_{ik}=h^{*}$. It is an arbitrage equilibrium [19] where the agents do not have any incentive to switch states anymore, as all states provide the same effective utility $h^{*}$. Thus, the maximization of ϕ and $h_{ik}=h^{*}$ are exactly equivalent criteria (if ϕ(x) is strictly concave), and both specify the same outcome, namely, an arbitrage equilibrium.

We are keenly aware, of course, of the simplifications we have made to make the analysis analytically tractable. We realize that our bird-like agents are not real birds, and our models and simulations are not real biological systems. They are stylized ideal systems formulated in the spirit of similar ideal systems in statistical mechanics, as noted. Despite such an ideal approximation, our results nevertheless suggest intriguing possibilities for real biological entities that need to be explored further.

### 2.1. Agent’s Utility: Position Dependence

Our goal is to develop a simple model of the effective utility ($h_{ik}$) of our bird-like agent *i* (which we call *Garud* to differentiate it from the agent *Boid* of the Reynolds model) in the state *k*. We want the model to be an appropriate coarse-grained description of the system that can make useful predictions not restricted by system-specific nuances. We have tried, deliberately, to keep the model as simple as possible without losing key insights and relevance to empirical phenomena. One can add more complexity, such as cooperation among agents or environmental conditions, as and when desired later on. Again, what we are aiming for is the equivalent of the ideal gas model or the Ising model for flocking.

Flocking dynamics is modeled by describing the time evolution of the position $r_{i}$ of the ith agent and its velocity $v_{i}$. Therefore, the state of the ith agent is specified by its position $r_{i}$ and velocity $v_{i}$. Since the index *i* itself now denotes the state, we do not need *k* as a separate index and therefore drop it. The state of the flock at any given time is specified by specifying $r_{i}$ and $v_{i}$ for all agents. When the agents move at a constant speed $v_{0}$, the state of the system is then determined by the set of agents’ positions and velocity directions or orientations ${\left\{r_{i},s_{i}\right\}}_{i=1}^{N}$, where *N* is the total number of agents.

An agent *i* is said to have affected an agent *j* if *j* is in the neighborhood of *i*, $N^{i}$. The neighborhood $N^{i}$ of *i* is defined by a matrix whose elements are $n_{ij}$, where

(2) $$\begin{array}{c} n_{ij}=\left\{\begin{array}{cc} 1 & \mathrm{if}\;j\;\mathrm{is}\;a\;\mathrm{neighbor}\;\mathrm{of}\;i\\ 0 & \mathrm{otherwise} \end{array}\right. \end{array}$$

The span of the neighborhood is specified in terms of the absolute distance between *i* and *j*, and a size parameter $r_{0}$ [11,25], such that

(3) $$\begin{array}{c} n_{ij}=\left\{\begin{array}{cc} 1 & |r_{i}\left(t\right)-r_{j}\left(t\right)|<r_{0}\\ 0 & \mathrm{otherwise} \end{array}\right. \end{array}$$

As seen from Equation (3), we consider agent *i* to be its own neighbor. One can also define a neighborhood in terms of a fixed topology of nearest neighbors [12,13,14], but we do not use this specification in this study. It follows that the number of neighbors of an agent *i* is given by $n_{i}=\sum_{j}n_{ij}$.

The effective utility $h_{i}$ is a model of the cost–benefit trade-offs that the agents use to make decisions dynamically as they maneuver to increase their utilities. There are four terms that contribute to the effective utility of an agent in state *i*: (i) utility of cohesion, (ii) disutility of congestion, (iii) utility of alignment, and (iv) disutility of competition. We believe that this structure is fairly general, capturing the essence of a wide variety of herding phenomena. The first three are similar to the three rules of agent behavior in the Reynolds model: (i) rule of cohesion, (ii) rule of separation, and (iii) rule of alignment. Our fourth term is unique and is not present explicitly in the Reynolds or the Vicsek models. As we explain below, this term corresponds to an *entropic restlessness* of the agents.

We consider the ith agent’s position in the frame of reference of the center of mass of its neighborhood. The utility of *cohesion* for the ith agent is proportional to its number of neighbors, $n_{i}$. However, this benefit comes at the *cost of congestion*, the disutility of congestion. The trade-off between the two terms, the cost–benefit trade-off, results in an inverted-U profile, which, following Venkatasubramanian [17], can be modeled as,

(4) $$h_{r}^{\left(i\right)}=\alpha n_{i}-\beta n_{i}^{2}$$

where $h_{r}^{\left(i\right)}$ is the position component of the utility for the ith agent; α,β>0. For many things in life, the cost–benefit trade-off is in the form of an inverted-U curve. Consider, for example, taking some medicine to cure an ailment. If one is suffering from a severe headache, taking one tablet might make you feel a little better, but taking two might help more. This does not mean that taking ten would help a lot more! It could actually make one sicker, triggering a whole set of more serious problems. As the dosage increases beyond a critical point, the benefit begins to go down, sometimes dramatically. This is so because the cost of the treatment (in the form of negative side effects) begins to exceed the benefit. Equation (4) captures and models this essential and near-universal trait in most things in life.

Note that the positional dependence is accounted for in the computation of $n_{i}$. Given a configuration of all agents $\left\{r_{i}\right\}$, the neighborhood of the ith agent is defined by the parameter $r_{0}$, where if the jth agent is within this radius, then it is considered a neighbor.

This, in turn, identifies a direction of increased utility, which is given by,

(5) $$\frac{\partial h_{r}^{\left(i\right)}}{\partial r_{i}}=\alpha \frac{\partial n_{i}}{\partial r_{i}}-2\beta n_{i}\frac{\partial n_{i}}{\partial r_{i}}$$

$\frac{\partial n_{i}}{\partial r_{i}}$ is dependent on the agents in the perimeter of the neighborhood of the reference agent *i*.

### 2.2. Agent’s Utility: Velocity Dependence

The utility of an agent is also dependent on the velocity of its neighbors in that the agent attempts to match the orientation with its neighboring agents. This utility of *alignment* ($h_{v}^{\left(i\right)}$) can be written as (γ>0),

(6) $$h_{v}^{\left(i\right)}=\gamma \sum_{j}n_{ij}\frac{v_{i}}{|v_{i}|}\cdot \frac{v_{j}}{|v_{j}|}$$

The utility of the ith agent, then, depends on the orientation of the other agents in its neighborhood, i.e., $s_{i}\cdot s_{j}$, where *j* is a neighbor of agent *i*. This gives the alignment utility for the ith agent as,

(7) $$h_{v}^{\left(i\right)}=\gamma \sum_{j}n_{ij}s_{i}\cdot s_{j}$$

where $n_{ij}$ shows if the jth agent is a neighbor of agent *i*, i.e.,

(8) $$\begin{array}{c} n_{ij}=\left\{\begin{array}{cc} 1 & |r_{i}\left(t\right)-r_{j}\left(t\right)|<r_{0}\\ 0 & \mathrm{otherwise} \end{array}\right. \end{array}$$

If each agent is perfectly aligned with its neighbors, this utility component is maximal, whereas if they are oriented in the opposite direction, this is minimal. Therefore, the agents prefer to be aligned. This gives the ith agent an arbitrage opportunity to adjust its velocity vector toward this direction to increase its utility. This opportunity for increasing its utility generates a self-propelled force on the ith agent. If the ith agent is not aligned with its neighbors, this direction of increased utility is given by,

(9) $$\frac{\partial h_{v}^{\left(i\right)}}{\partial s_{i}}=\gamma \sum_{j}n_{ij}s_{j}$$

### 2.3. Agent’s Effective Utility

There is the fourth utility component remaining to be considered. As noted, this is not stated explicitly in the three rules of the boids. However, it is implied because it is assumed that the boids have to be constantly moving. So, as an agent incessantly jockeys and moves for better positions and orientations, its ability to do so is hampered by the *competition* from other agents in its neighborhood that are also trying to do the same. As Venkatasubramanian explains [17], this disutility of competition can be modeled as $-\delta \ln n_{i}$. This term, when integrated to obtain the potential ϕ(x), leads to entropy in statistical mechanics. Thus, maximizing the potential ϕ(x) is equivalent to maximizing entropy. This is why we call this term *entropic restlessness*. In order to increase the effective utility $h_{i}$, the agents try to minimize $\ln n_{i}$ (because of the negative sign in front of it). Thus, the agents try to move to other states that have lower $n_{i}$. Since they are constantly doing this, this leads to *restless* behavior and constant movement. For more details, the reader is referred to Venkatasubramanian [17,20].

Now, by combining all these components, we arrive at the *effective utility* for the ith agent given by

(10) $$\begin{array}{cc} h_{i} & =\alpha n_{i}-\beta n_{i}^{2}+\gamma \sum_{j}n_{ij}s_{i}\cdot s_{j}-\delta \ln n_{i}\\ h_{i} & =\alpha n_{i}-\beta n_{i}^{2}+\gamma n_{i}l_{i}-\delta \ln n_{i} \end{array}$$

where $l_{i}$ = $\frac{1}{n_{i}}\sum_{j}n_{ij}s_{i}\cdot s_{j}$ is the average alignment of agent *i*. Without any loss of generality, δ can be assumed to be 1 and will be assumed as such for the rest of this paper. When α,β,γ=0, the agents do not have any preferences and hence fly around randomly. This is what is captured by the remaining $-\ln n_{i}$ term, i.e., *entropic restlessness*. These four components are summarized in Table 1 for the convenience of the reader.

Statistical teleodynamics, via potential game theory, proves that the self-organizing dynamics of the agents would eventually result in *arbitrage equilibrium*, where ϕ(x) is maximized, and the effective utilities of all the agents are the same, i.e., $h_{i}=h^{*}$. This proof is a well-known result, and our reader is referred to the literature for more details [15,16,18,20,22]. Thus, this answers the first question we raised in Section 1 in the affirmative. The answer to the second question is that it is an arbitrage equilibrium where the effective utilities of all the agents are equal.

In the next section, we discuss our agent-based simulation results that confirm this prediction. We also answer the remaining two questions. Although our framework is somewhat similar to the reward-driven agent-based model of Durve et al. [26] or of Katare et al. [27], it is entirely different in the conceptual and mathematical formulation. The other approaches do not use potential game theory and arbitrage equilibrium in their formulations, which are important distinctions.

## 3. Results

For illustrative purposes, we show that our formulation leads to flocking behavior that is similar to that of the Reynolds model. Again, we wish to remind the reader that our objective is not to mimic Reynolds’s model. Our objective is to demonstrate an entirely different modeling framework of self-organization that is founded on goal-driven arbitrage behavior and utility maximization by biological agents.

The Reynolds model for the ith agent is given by the equation (see Supplementary Information, Equation (11)),

(11) $$\begin{array}{c} v_{i}\left(t+\Delta t\right)=v_{i}\left(t\right)+\left(\frac{a}{n_{i}}\sum_{j}n_{ij}\left(r_{j}-r_{i}\right)+b\sum_{j}n_{ij}\left(r_{i}-r_{j}\right)+\frac{c}{n_{i}}\sum_{j}n_{ij}\left(v_{j}-v_{i}\right)+\eta \left(t\right)\right)\Delta t \end{array}$$

where a,b, and *c* are parameters corresponding to the rules of cohesion, separation, and alignment, respectively. Parameter η(t) is the uncorrelated noise in the agent’s velocity. The time-scale, Δt, in Equation (11), can be subsumed in a,b,c. For the simulation details, the reader is referred to Methodology in Section 5 below. If the agents are flying randomly, without pursuing utility, then this base case corresponds to α,β,γ,=0;δ=1. This result is discussed in Section S4.

**Figure 1:**
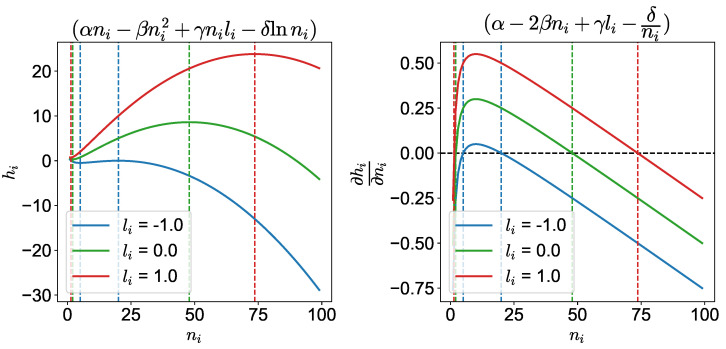
Effective utility and its derivative as a function of the number of neighbors, $n_{i}$, for different values of alignment $l_{i}$ (α,β,γ,δ=0.5,0.005,0.25,1). There are two points where the derivative of the effective utility is zero for different alignments.

For the other cases, the effective utility function in Equation (10) is plotted in Figure 1 in terms of the number of neighbors of agent *i* ($n_{i}$) for different alignments ($l_{i}$) for a given set of α,β,γ. We see that there are two values of ${\hat{n}}_{i}$ (${\hat{n}}_{-}$ and ${\hat{n}}_{+}$) where the gradient of utility, for a given value of alignment, is zero. These correspond to the optimal flock size ${\hat{n}}_{i}$ for maximum effective utility ($h_{i}$). These values are determined by (see Supplementary Information, Section S1).

(12) $${\hat{n}}_{i}=\frac{\left(\alpha +\gamma l_{i}\right)\mp \sqrt{{\left(\alpha +\gamma l_{i}\right)}^{2}-8\beta \delta }}{4\beta }$$

In Figure 1, at the lower value (${\hat{n}}_{-}$), any deviation in ${\hat{n}}_{-}$ increases the utility of the agent and hence leads to an unstable point. However, for the higher point (${\hat{n}}_{+}$), we see that any deviation reduces the agent’s utility. Therefore, this leads to a stable point, as any deviation would bring an agent back to the higher utility state. Therefore, this is the point an agent will try to reach to maximize its utility. For example, for the red curve, this would correspond to the point where ${\hat{n}}_{+}=73.6$. However, despite this point’s stability, an agent will not be able to stay there indefinitely, as the other agents in its neighborhood are competing for its position by constantly changing their positions and orientations in their flights. Therefore, the ith agent would be fluctuating around this point. This behavior is similar to what is seen in other systems around the *spinodal* and *binodal* points [20]. While the individual agents would prefer to be at their maximum utility point (the spinodal point), the system as a whole is driven by the competition among the agents to the binodal point where the potential ϕ(x) is maximized. Thus, the agents wander around in phase space between these two points at equilibrium, as shown in the following.

**Figure 2:**
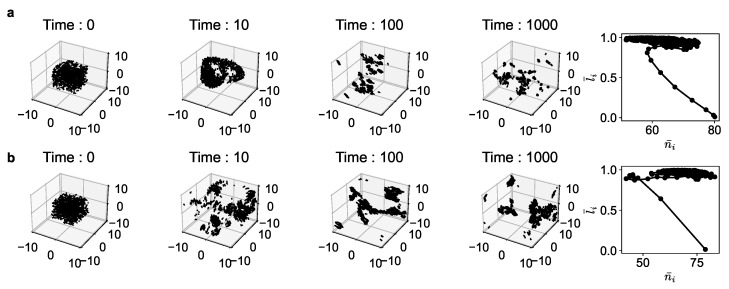
Trajectory of the agents and the corresponding phase portrait in $n_{i}-l_{i}$ space for the average number of neighbors of each individual agent during the course of the simulation, corresponding to (**a**) Reynolds’ boids for a=0.5,b=0.01,c=0.5 and (**b**) Utility-driven agents for α=0.5,β=0.01,γ=0.25,δ=1.

**Figure 3:**
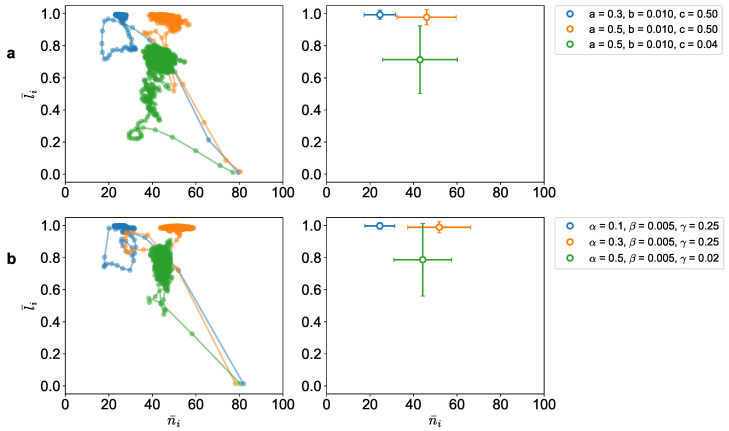
Trajectory of the average of number of neighbors of each agent and average alignment of the agents in the $n_{i}$, $l_{i}$ phase space, and corresponding estimated averages for the (**a**) Reynolds’ model (**b**) Utility-driven model (δ=1).

In Figure 2 and Figure 3, we show the simulation results of both the Reynolds model and our utility-driven agent model. The results are shown for different parameter values of (a,b,c) and (α,β,γ). From the simulations, we obtain a set of position and velocity values $\left\{r_{i},v_{i}\right\}$ of each agent *i* at every time-step. Once this is obtained, we extract the features $n_{i}$ and $l_{i}$ for all the agents. This, in turn, is used to compute the average number of neighbors $\bar{n}$ and average alignment $\bar{l}$ for the entire population for all time points (Figure 2).

Figure 2 shows the snapshots at different time points of the evolution in 3D space and the $n_{i}-l_{i}$ phase space. While the exact dynamics, the exact configuration of the population, and the time scale of evolution of these two models cannot be the same (and that is not the aim, either), we observe that the qualitative patterns of collective behavior are very similar in both cases. In particular, we see that all agents gravitate toward a certain region in the $n_{i}-l_{i}$ phase space for both models. They both start at the lower far right point (where the average alignment is near zero as the agents are all randomly oriented initially and closely packed) and evolve toward the upper center-right region in black. We notice a qualitative match of both trajectories.

Furthermore, we can also see a quantitative similarity between the two models for specific parameters (Figure 3). Figure 3a shows the phase space for the Reynolds model, and Figure 3b shows the same for the utility-driven model. In both models, we notice similar features of evolution toward the arbitrage equilibrium states, starting from the lower right point at time t=0 to ending in the colored regions, where the average number of neighbors and the average alignment fall in similar corresponding regions. The plots on the right show the average alignment and average number of neighbors of the agents in the last 100 time-steps.

Although both models exhibit similar collective behaviors, it is not apparent, however, from the three rules of the Reynolds model that its dynamics would result in an equilibrium state in the $n_{i}-l_{i}$ phase space. This is an important difference between the two approaches. Since the potential game formulation predicts an arbitrage equilibrium outcome (thus answering questions (i) and (ii)), it is clear right from the beginning where in the phase space the system is going to end up (and hence answer the question (iii)). We can therefore make a quantitative prediction about the optimal flock size ${\hat{n}}_{+}$ and the corresponding effective utility $\hat{h}$ values at equilibrium. From Equation (12), we can calculate the optimal flock size ${\hat{n}}_{+}$ for the three configurations shown in Figure 3b. We compare these predictions with the observed values from the simulations (Table 2). As we can see, the predictions are consistent with the simulation results. As noted above, given the competitive dynamics of the *garuds*, the agents are not able to stay with the optimal flock size but keep fluctuating around it as the standard deviation metrics indicate.

This ability to predict the final outcome of the collective behavior of the population, given the individual agent-level properties captured in the utility function $h_{i}$, is an important defining feature and strength of the statistical teleodynamics framework. An additional characteristic is the ability to prove the stability of the final outcome, as we discuss in Section 3.1, and answer our fourth question [15,17].

**Figure 4:**
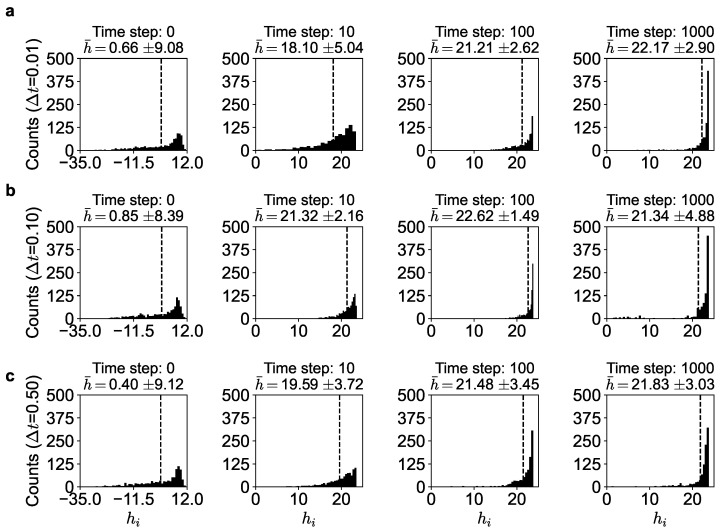
Histogram of the utility ($h_{i}$) of the agents for α,β,γ,δ=0.5,0.005,0.25,1 corresponding to (**a**) Δt=0.01, (**b**) Δt=0.1 and (**c**) Δt=0.5. Dashed line shows the average utility at a particular time.

We also ran the simulations for different time-step sizes of Δt=0.01,0.1,0.5 in Equation (11) to understand the dynamics of the evolution better. Note in Figure 4 that at the start (t=0), the utilities of all the agents are spread out, with many having negative utility values and the average utility being ($\bar{h}$) low. However, as the dynamics evolves, every agent tries to increase its utility by maneuvering to a better neighborhood and better orientation, the distribution becomes narrower, the average utility keeps increasing, and it reaches a near-maximum value ($\bar{h}=22.17\pm 2.90$ in Figure 4a) and fluctuates around it. Note that this is around the maximum theoretical value of about 23.8 (given by Equation (10)), where the histogram peaks. This suggests that nearly all the agents have similar effective utilities asymptotically, approaching the maximum. This, of course, is the *arbitrage equilibrium* outcome predicted by the theory (see also Supplementary information, Figure S3). The agents do not converge exactly on $h^{*}$ but fluctuate around it because of the stochastic dynamics. This is also seen in Table 3 where nearly the top 10 % of the agents at a particular time-step are very close to the maximum utility value. In fact, the top 50% of the agents have an average utility of greater than 23.

At the risk of belaboring this point, we reiterate, in order to be clear, that as noted in Figure 1 and Figure 4, the self-organized dynamics strives toward maximum utility. From Equation (10), for a given set of α, β, γ, and δ values, we can determine the arbitrage equilibrium state defined by $\hat{h}$ and ${\hat{n}}_{+}$. For α=0.5, β=0.005, γ=0.25, and δ=1, we have $\hat{h}=23.8$ and ${\hat{n}}_{+}=73.6$. This is predicted by our model analytically. We see this confirmed in the simulation as shown in Figure 4 and Table 1. Due to the inherent stochastic dynamical nature of the agents’ movements, the agents cannot stay at the optimum even after finding it. They keep moving around about it. We see this from the table: we see that about 50% of the agents enjoy a utility of 23.6, which is very close to the theoretical maximum of 23.8 (for a step size of 0.01).

This arbitrage equilibrium state is unique only if the potential ϕ(x) is strictly concave [22]. For our boids-like agents, this is not the case in general, as the concavity would depend on α, β, and γ having some particular values. So, for the typical case where ϕ(x) is not concave, there could be multiple equilibrium configurations of the agents. Thus, instead of an equilibrium *point* in the $n_{i}-l_{i}$ phase space, one has an equilibrium *region* in general. In other words, invoking a terminology from chaos and nonlinear dynamics, there is a *basin of attraction* in the phase space where the agents finally settle in and fly around. We see this in Figure 2 and Figure 3—the basins are the colored regions.

### 3.1. Stability of the Arbitrage Equilibrium

We can answer our fourth question and determine the stability of this equilibrium by performing a Lyapunov stability analysis [15,17]. A Lyapunov function *V* is a continuously differentiable function that takes positive values everywhere except at the equilibrium point (i.e., *V* is positive definite), and it decreases (or is nonincreasing) along every trajectory traversed by the dynamical system ($\dot{V}$ is negative definite or negative semidefinite). A dynamical system is locally stable at equilibrium if $\dot{V}$ is negative semidefinite and is asymptotically stable if $\dot{V}$ is negative definite.

Following Venkatasubramanian [17], we identify our Lyapunov function V(n)

(13) $$\begin{array}{c} V\left(n\right)=\phi^{*}\left(n^{*}\right)-\phi \left(n\right) \end{array}$$

where $\phi^{*}$ is the potential at the Nash equilibrium (recall that $\phi^{*}$ is at its maximum at NE), ϕ(n) is the potential at any other state, and n is the vector of the neighbors ($n_{i}$) of all agents. Note that V(n) has the desirable properties we seek: (i) $V(n^{*}$) = 0 at NE and V(n)> 0 elsewhere, i.e., V(n) is positive definite; (ii) since ϕ(n) increases as it approaches the maximum, V(n) decreases with time, and hence, it is easy to see that $\dot{V}$ is negative definite. Therefore, the arbitrage equilibrium is not only stable but also *asymptotically stable.*

**Figure 5:**
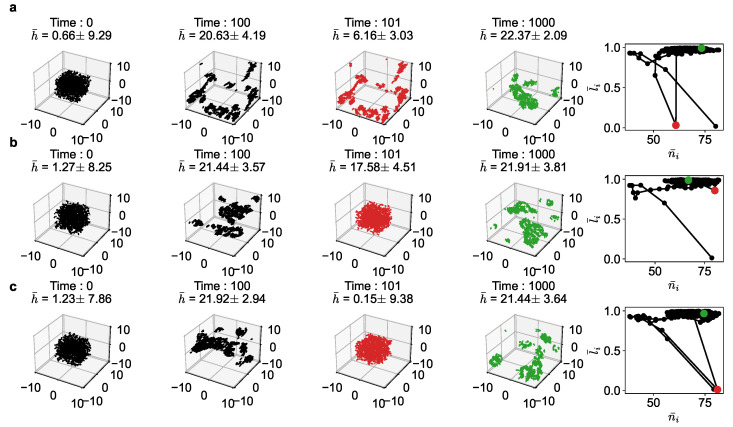
The trajectory of the agents as a function of time and the corresponding phase portraits for α,β,γ,δ=0.5,0.005,0.25,1 for stability analysis case studies (**a**) Disturbance 1, (**b**) Disturbance 2, and (**c**) Disturbance 3. The disturbances occur after equilibrium at a time-step of 101 (in red). The configuration at the end of the simulation is also shown is shown (in green).

Our simulation results confirm this theoretical prediction (see Figure 5). After the agents population reached equilibrium, we disturbed the equilibrium state by randomly changing the positions and/or velocities of the agents. The simulation is then continued from the new disturbed far-from-equilibrium state. We conducted experiments with three kinds of disturbances:

- **Disturbance 1**: *Velocity disturbance*, where each agent’s velocity is changed to a random orientation and magnitude.
- **Disturbance 2**: *Position disturbance*, where each agent’s position is randomly changed.
- **Disturbance 3**: *Position and velocity disturbance*, where both position and velocity vectors are changed.

As seen in Figure 5, after the 100th time-step, when the population had reached equilibrium, we introduced these disturbances. The 101st time-step shows in red color the new far-from equilibrium states, where the average utility (*h*) has dropped considerably. In all cases, the population recovers quickly, typically in another 100 time-steps or so, to reach the original equilibrium region (shown in green). The figure shows the disturbance (red) and recovery (green) in both the 3D space and the phase space.

In Figure 5a, as the velocities are randomized at the 101st time-step, the alignment goes down to 0, but it recovers to the original equilibrium quickly. In Figure 5b, as the new configuration corresponds to a similar value of an average number of neighbors as before, the disturbance is not that much. Note that the drop in average utility is small. In Figure 5c, we see that this disturbance is huge, pushing the configuration close to the original random state. Still, the population is able to recover to the arbitrage equilibrium quickly.

This shows that the arbitrage equilibrium region is not only stable but asymptotically stable. That is, the agents’ flocking configuration is resilient and self-healing. Given the speed of the recovery, it could possibly be exponentially stable, but we have not proved this analytically here. It is interesting to observe that this result is similar to that of the income-game dynamics [15,17].

## 4. Conclusions

For three centuries, we have known that there are constants of motion, such as energy and momentum, for passive matter. Nevertheless, it comes as a surprise to discover that the dynamics of active matter populations could also have an invariant, namely, the effective utility. However, the role of invariance here is different from its role in classical mechanics. The constants of motion, such as energy and momentum, are conserved, whereas the effective utility is not. The role of this invariance is more like that of set-point tracking and disturbance rejection in feedback control systems [28,29]. The system, i.e., the agents’ population, adjusts itself dynamically and continually, in a feedback control-like manner, to maintain its overall maximum potential ϕ(x).

It is important to emphasize, however, that this control action is decentralized as opposed to the typical centralized control system in many engineering applications. The agents individually self-organize, adapt, and dynamically course correct to offset the negative impact on their effective utilities by other agents or other external sources of disturbance. The population as a whole stochastically evolves toward the stable basin of attraction in the phase space in a self-organized and distributed-control fashion.

The same mathematical framework has been demonstrated for other dynamical systems, as summarized in Table 4, in biology [20], ecology [20], sociology [20], and economics [15,17,19] to predict emergent phenomena via self-organization. As Venkatasubramanian et al. [20] showed, the emergence of the exponential energy (i.e., Boltzmann) distribution for gas molecules can be modeled by the effective utility

(14) $$h_{i}=-\beta \ln E_{i}-\ln n_{i}.$$

Similarly, they showed [20] that for biological systems, the cost–benefit trade-off in effective utility $h_{i}$ for bacterial chemotaxis can be modeled by

(15) $$h_{i}=\alpha c_{i}-\ln n_{i}$$

where the first term is the benefit derived from a resource ($c_{i}$, α>0) and the second is the cost of competition as modeled in Equation (10).

In a similar vein, the emergence of ant craters can be modeled by

(16) $$h_{i}=b-\frac{\omega r_{i}^{a}}{a}-\ln n_{i}.$$

where the first term (*b*) is the benefit of having a nest for an ant, the second term is the cost of work performed when carrying the sand grains out to build the nest, and the last term is again the cost of competition as before.

The same study showed how the Schelling game-like segregation dynamics in sociology can be modeled by

(17) $$\begin{array}{c} h_{i}=\eta n_{i}-\xi n_{i}^{2}+\ln\left(H-n_{i}\right)-\ln n_{i} \end{array}$$

where the first term is the benefit of the community of neighbors, the second is the congestion cost of such neighbors, the third is the benefit of relocation options, and the last is again the cost of the competition.

In economics, the emergence of an income distribution can be modeled by

(18) $$h_{i}=\alpha \ln S_{i}-\beta {\left(\ln S_{i}\right)}^{2}-\ln n_{i}.$$

where the first term is the benefit of income, the second is the cost of work expended to earn this income, and the last is again the cost of the competition.

By comparing Equations (14) through (18) with equation Equation (10) for *garuds* (δ=1),

$$h_{i}=\alpha n_{i}-\beta n_{i}^{2}+\gamma n_{i}l_{i}-\ln n_{i}$$

We observe a certain universality in the structure of the effective utility functions in different domains. They are all based on cost–benefit trade-offs, but the actual nature of the benefits and costs depends on the details of the specific domain, as one would expect. Thus, we see that the same conceptual and mathematical framework is able to predict and explain the emergence of spontaneous order via self-organization to reach arbitrage equilibrium in dynamical systems in physics, biology, ecology, sociology, and economics.

These results suggest that the pursuit of maximum utility or survival fitness could be a universal self-organizing mechanism. In the case of birds, the incessant search for improving survival fitness occurs in the three-dimensional physical space, with the birds trying to move to a better location. In biology, in general, the search for improving one’s fitness occurs in the design space of genetic features. Here, the mutation and crossover operations facilitate the movements in the feature space, such that an agent improves itself genetically via Darwinian evolution to increase its utility, i.e., the survival fitness. In economics, on the other hand, agents search in the products and/or services space so that they can offer better products/services to improve their economic survival fitness in a competitive marketplace. Thus, this mechanism is essentially the same as Adam Smith’s *invisible hand*. In all these different domains, every agent is pursuing its own self-interest to increase its own $h_{i}$, but a stable collective order emerges, nevertheless, spontaneously via self-organization.

Thus, our theory shows an important result that these so-called out-of-equilibrium systems are actually in equilibrium, an *arbitrage* equilibrium. Just as systems can be in mechanical equilibrium when forces or pressures are equal or in thermal equilibrium when temperatures are equal, or in phase equilibrium when the chemical potentials are equal, we have active matter systems in arbitrage equilibrium when the utilities are equal.

As noted, we do realize that our bird-like agents are not real birds, and we are not implying that real birds make decisions along the lines of our model necessarily. Nevertheless, our results suggest interesting possibilities for real biological entities that need to be studied further. What we have here is for ideal systems, of course, similar to the ideal gas or the Ising model in thermodynamics. Just as real gases and liquids do not behave exactly like their ideal versions in statistical thermodynamics, we do not expect real biological systems (or economic or ecological systems) to behave like their ideal counterparts in statistical teleodynamics. Nevertheless, the ideal versions serve as useful starting and reference points as we develop more comprehensive models of active matter systems. The next steps would involve examining and learning how real-world biological systems behave compared to their ideal versions. This would, of course, necessitate several modifications to the ideal models.

## 5. Methods

We created a simulation of 1000 agents in a periodic box of dimensions 20×20×20, where each agent’s neighborhood is a sphere with radius $r_{0}=3$. Each agent starts at a random location and orientation inside a 10×10×10 block. The speed of each agent is limited between 0.5 and 1. The update algorithm works similar to Reynolds’ agent’s update, except the force is driven by the numerical estimates of the direction of increased utility (additively based on position and velocity). An additional noise is also added to the velocity update strategy similar to Reynolds’ model to capture the erroneous strategies of velocity update for each agent. This is given by a noise parameter (0.01, unless specified) times the magnitude of the velocity. The noise indicates that an agent does not make perfect choices in updating its velocity. It is to be noted that there are different ways of realizing the dynamics of the system, which may result in distinctive dynamic behaviors. The goal, however, is to show that they eventually reach the same game-theoretic equilibrium.

## Figures and Tables

**Table 1:** Components of the effective utility $h_{i}$.

Utility of Cohesion	$\alpha n_{i}$	Benefit of having $n_{i}$ neighbors
Disutility of Congestion	$-\beta n_{i}^{2}$	Cost of crowding by $n_{i}$ neighbors
Utility of Alignment	$\gamma \sum_{j}n_{ij}s_{i}\cdot s_{j}$	Benefit of being aligned with the neighbors
Disutility of Competition	$-\ln n_{i}$	Cost of competition from the $n_{i}$ neighbors
		Entropic restlessness

**Table 2:** Optimal flock sizes at equilibrium.

Configuration	Predicted Flock Size	Observed Flock Size
α=0.1,β=0.005,γ=0.25	31.9	25.3 ± 8.0
α=0.3,β=0.005,γ=0.25	53.1	50.1 ± 16.0
α=0.5,β=0.005,γ=0.02	50.0	44.5 ± 13.4

**Table 3:** Utility of different percentiles of the agents at the 1000th time-step corresponding to Figure 4.

Population	Time-Step Size, Δt	Average Utility
Top 1%	0.01	23.79
	0.1	23.80
	0.5	23.76
Top 1–10%	0.01	23.77
	0.1	23.78
	0.5	23.68
Top 10–50%	0.01	23.60
	0.1	23.51
	0.5	23.35
Top 50–75%	0.01	22.83
	0.1	22.64
	0.5	22.58
Bottom 50%	0.01	18.59
	0.1	15.59
	0.5	17.91

**Table 4:** Utility function in different domains.

Domain	System	Utility Function ($h_{i}$)
Physics	Thermodynamic game	$-\beta \ln E_{i}-\ln n_{i}$
Biology	Bacterial chemotaxis	$\alpha c_{i}-\ln n_{i}$
Ecology	Ant crater formation	$b-\frac{\omega r_{i}^{a}}{a}-\ln n_{i}$
Sociology	Segregation dynamics	$\eta n_{i}-\xi n_{i}^{2}+\ln\left(H-n_{i}\right)-\ln n_{i}$
Economics	Income game	$\alpha \ln S_{i}-\beta {\left(\ln S_{i}\right)}^{2}-\ln n_{i}$
Ecology	Garuds game	$\alpha n_{i}-\beta n_{i}^{2}+\gamma n_{i}l_{i}-\ln n_{i}$

## Data Availability

The data is simulated using the model shown in Supplementary Information.

## Supplementary Materials

The following supporting information can be downloaded at: https://www.mdpi.com/article/10.3390/e25071043/s1.
