# Supplementary material for: Arbitrage Equilibrium, Invariance, and the Emergence of Spontaneous Order in the Dynamics of Bird-like Agents
Source: Entropy (Basel). 2023 Jul 11;25(7):1043. doi: 10.3390/e25071043 (PMC10378221; doi:10.3390/e25071043)
Supplement: Supplementary file 1 [file entropy-25-01043-s001.zip › entropy-2436072-Supplementary.pdf]

Supplementary Materials: Arbitrage Equilibrium, Invariance, and the Emergence of Spontaneous Order in the Dynamics of Bird-like Agents: Supplementary Information

Abhishek Sivaram[1] and Venkat Venkatasubramanian[2,*]

S1. Derivation of the optimum

At the arbitrage equilibrium, all agents have the same effective utility, h^*, i.e.,

$$h_i = \alpha n_i - \beta n_i^2 + \gamma n_i l_i - \delta \ln n_i = h^*$$

Now, h_i is maximum at two n_i values where the gradient is zero, given by,

$$\begin{aligned}
\frac{\partial h_i}{\partial n_i} &= \alpha - 2\beta n_i + \gamma l_i - \frac{\delta}{n_i} = 0 \\
\implies -2\beta n_i^2 + (\alpha + \gamma l_i) n_i - \delta &= 0 \\
n_{\mp} &= \frac{(\alpha + \gamma l_i) \mp \sqrt{(\alpha + \gamma l_i)^2 - 8\beta\delta}}{4\beta}
\end{aligned}$$

Note that n_- is an unstable point as any deviation in the number of neighbors would result in increasing utility, thereby causing the agent to move away from there. On the other hand, n_+ is a stable point because any deviation would decrease the utility, thereby causing the agent to return to its original state.

S2. Dynamical Models of Flocking

Regarding the discussion in Section 2, the net effect of the three forces in the Reynolds model on the velocity of the ith boid is modeled by the equation,

$$\begin{aligned}
\mathbf{v}_i(t+1) = \mathbf{v}_i(t) + a(\mathbf{r}_{c,i} - \mathbf{r}_i) + b\sum_j n_{ij}(\mathbf{r}_i - \mathbf{r}_j) \\
+ c(\mathbf{v}_{c,i} - \mathbf{v}_i) + \boldsymbol{\eta}(t)
\end{aligned} \tag{S1}$$

where $a, b,$ and c are parameters corresponding to the *rule of cohesion*, *rule of separation*, and *rule of alignment*, respectively, $\mathbf{v}_{c,i}$ is the average velocity of the neighbors of i, and $\mathbf{r}_{c,i}$ is the center of the neighborhood as perceived by the agent i [1]. These are given by

$$\begin{aligned}
\mathbf{v}_{c,i} &= \frac{1}{n_i}\sum_{j\in\mathcal{N}^i} \mathbf{v}_j = \frac{1}{n_i}\sum_j n_{ij}\mathbf{v}_j \\
\mathbf{r}_{c,i} &= \frac{1}{n_i}\sum_j n_{ij}\mathbf{r}_j
\end{aligned}$$

Parameter $\boldsymbol{\eta}$ is the uncorrelated noise in the agent's velocity. Substituting the average velocity of the neighbors and the center of the neighborhood as perceived by the agent i, Eq. (S1) can be simplified to give Eq. (S2),

$$\begin{aligned}\mathbf{v}_i(t+1) = \mathbf{v}_i(t) \quad & + \frac{a}{n_i}\sum_j n_{ij}(\mathbf{r}_j - \mathbf{r}_i) + b\sum_j n_{ij}(\mathbf{r}_i - \mathbf{r}_j) \\ & + \frac{c}{n_i}\sum_j n_{ij}(\mathbf{v}_j - \mathbf{v}_i) + \boldsymbol{\eta}(t)\end{aligned} \tag{S2}$$

In general, we can write the above equation as shown in Eq. (S3),

$$\mathbf{v}_i(t+\Delta t) = \mathbf{v}_i(t) + \left(\frac{a}{n_i}\sum_j n_{ij}(\mathbf{r}_j - \mathbf{r}_i) + b\sum_j n_{ij}(\mathbf{r}_i - \mathbf{r}_j) + \frac{c}{n_i}\sum_j n_{ij}(\mathbf{v}_j - \mathbf{v}_i) + \boldsymbol{\eta}(t)\right)\Delta t \tag{S3}$$

for a time-step Δt. The time-scale Δt in Eq. (S3) can be subsumed by the parameters to give Eq. (S2)

The Vicsek model is a similar model where the velocity update is purely a function of the alignment of an agent with it's neighbors. The constant velocity dynamics is sometimes modified to include other pair-wise attraction-repulsion forces $\mathbf{f}_{ij}$ and is written as shown in [2],

$$\begin{aligned}\mathbf{r}_i(t+1) \quad & = \mathbf{r}_i(t) + \mathbf{v}_i(t) \\ \mathbf{v}_i(t+1) \quad & = v_0\Theta\left[\alpha\sum_j n_{ij}\mathbf{v}_j(t) + \beta\sum_j n_{ij}\mathbf{f}_{ij} + \eta_i(t)\right]\end{aligned}$$

where Θ scales the dynamics to a unit vector to ensure the constant speed of the individual agents.

Garud's forces The forces on the *garuds* can be established using the direction of increasing utility of each *garud*. This is a function of $\mathbf{r}_i$ and $\mathbf{s}_i$. Each garud reorients and changes its speed, so as to increase its utility.

We know that,

$$n_i = \sum_j n_{ij} = \sum_j \mathbb{1}(|\mathbf{r}_i - \mathbf{r}_j| < r_0)$$

$$\frac{\partial n_i}{\partial \mathbf{r}_i} = -\sum_j \frac{\delta(|\mathbf{r}_i - \mathbf{r}_j| - r_0)}{|\mathbf{r}_i - \mathbf{r}_j|}(\mathbf{r}_i - \mathbf{r}_j) = -\sum_j f_{ij}(\mathbf{r}_i - \mathbf{r}_j)$$

where f_{ij} establishes if the jth *garud* is at the perimeter of the neighborhood of the ith. It follows that the gradient of the utility with respect to $\mathbf{r}_i$, is given by,

$$\begin{aligned}\frac{\partial h_i}{\partial \mathbf{r}_i} = \quad & \alpha\frac{\partial n_i}{\partial \mathbf{r}_i} - 2\beta n_i\frac{\partial n_i}{\partial \mathbf{r}_i} + \gamma\sum_j\frac{\partial n_{ij}}{\partial \mathbf{r}_i}\mathbf{s}_i\cdot\mathbf{s}_j - \delta\frac{1}{n_i}\frac{\partial n_i}{\partial \mathbf{r}_i} \\ = \quad & \alpha\frac{\partial n_i}{\partial \mathbf{r}_i} - 2\beta n_i\frac{\partial n_i}{\partial \mathbf{r}_i} + \gamma\frac{\partial n_i}{\partial \mathbf{r}_i}l_i - \delta\frac{1}{n_i}\frac{\partial n_i}{\partial \mathbf{r}_i} \\ = \quad & -\alpha\sum_j f_{ij}(\mathbf{r}_i - \mathbf{r}_j) + 2\beta n_i\sum_j f_{ij}(\mathbf{r}_i - \mathbf{r}_j) \\ & -\gamma l_i\sum_j f_{ij}(\mathbf{r}_i - \mathbf{r}_j) + \frac{\delta}{n_i}\sum_j f_{ij}(\mathbf{r}_i - \mathbf{r}_j)\end{aligned}$$

Similarly, the gradient of the utility of the ith *garud* with respect to the orientation can be estimated to be,

$$\frac{\partial h_i}{\partial \mathbf{s}_i} = \gamma \sum_j n_{ij} \mathbf{s}_j$$

Depending on the position and velocity of the *garuds* $\{\mathbf{r}_i, \mathbf{v}_i\}$ each *garud* feels a force depending on these individual utility maximizing components. We assume this is additive, and the net force is given by,

$$\begin{aligned} \mathbf{F}_i = & \frac{\partial h_i}{\partial \mathbf{r}_i} + \frac{\partial h_i}{\partial \mathbf{s}_i} \\ = & -\alpha \sum_j f_{ij}(\mathbf{r}_i - \mathbf{r}_j) + 2\beta n_i \sum_j f_{ij}(\mathbf{r}_i - \mathbf{r}_j) \\ & -\gamma l_i \sum_j f_{ij}(\mathbf{r}_i - \mathbf{r}_j) + \gamma \sum_j n_{ij} \mathbf{s}_j + \frac{\delta}{n_i} \sum_j f_{ij}(\mathbf{r}_i - \mathbf{r}_j) \end{aligned}$$

This results in the velocity and position update rule given by,

$$\mathbf{v}_i(t + \Delta t) = \mathbf{v}_i(t) + \mathbf{F}_i(t)\Delta t + \boldsymbol{\eta}(t)\Delta t \quad \text{(S4)}$$

$$\mathbf{r}_i(t + \Delta t) = \mathbf{r}_i(t) + \mathbf{v}_i(t)\Delta t \quad \text{(S5)}$$

where $\boldsymbol{\eta}$ gives the error in estimation of the direction of increased utility.

S3. Base case: $\alpha, \beta, \gamma = 0$

Fig. S1 shows the case where α, β, γ are set to zero, so the agents are entropically driven (only $-\ln n_i$ component is driving the motion of the agents). In this case, the agents start with a high density and zero alignment (Fig. S1**a**) initial configuration and finally settle in a configuration where they have random positions and velocities (Fig. S1**b**). If each agent is randomly located in 3D space, the average number of neighbors for each agent is given by,

$$\bar{n}_i = \frac{N}{L^3} \left(\frac{4}{3} \pi r_0^3 \right) \quad \text{(S6)}$$

where $L = 20$ is the length of the domain, and $r_0 = 3$ is the size of the neighborhood. With a total of 1000 boids, this gives an estimate of the average number of neighbors of the ith agent as 14.1. The random alignment of the agent also gives the average alignment with neighbors as zero, as there is no incentive to increase the alignment. The simulation results confirm the theoretical expectations.

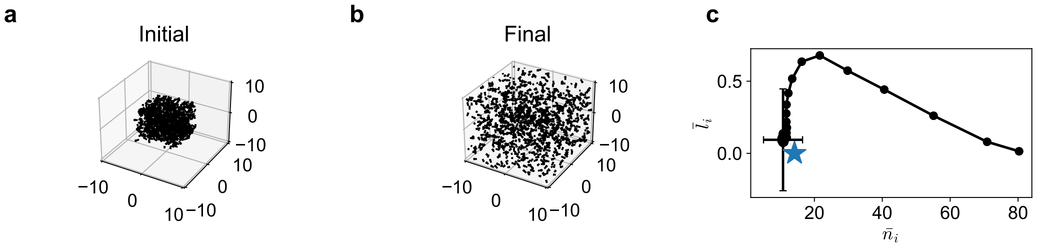

Figure S1. (**a**) Initial configuration of agents, (**b**) Final configuration, and (**c**) Phase space for $\alpha, \beta, \gamma = 0, 0, 0; \delta = 1$. The system is entropically driven. The star shows the theoretical average $\bar{n}_i$, $\bar{l}_i$ in the case where all the agents are randomly distributed ($\bar{n}_i$ = $\sim$14, $\bar{l}_i$=0).

S4. Noise trend

The noise parameter is also varied in the simulations. To reiterate, the noise-parameter dictates the magnitude of randomness in the velocity vector which is added to the ideal "direction" of increasing utility. As the noise keeps increasing, we see that the system tends more and more towards randomness characterized by random positions and velocities of agents (dashed lines in Fig. S2).

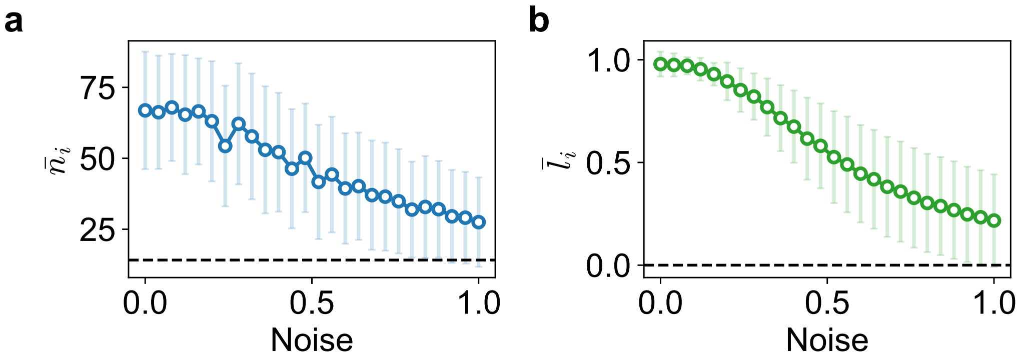

Figure S2. Neighbors (**a**) and alignment (**b**) dependence on noise parameter for utility parameters $\alpha, \beta, \gamma = 0.5, 0.005, 0.25$. We see that increasing the noise in decision-making results in the system tending more towards random behavior (dashed lines in **a** and **b**)

S5. Dynamics variation for different time step sizes

We also ran the simulation for different time step sizes of $\Delta t = 0.01, 0.1, 0.5$ in Eq. S3. Fig. S3 shows that the dynamics are quite different of the agents for the different step sizes. However, the corresponding phase plots suggest that irrespective of the different dynamics, the agents return to the same region in the phase space at equilibrium.

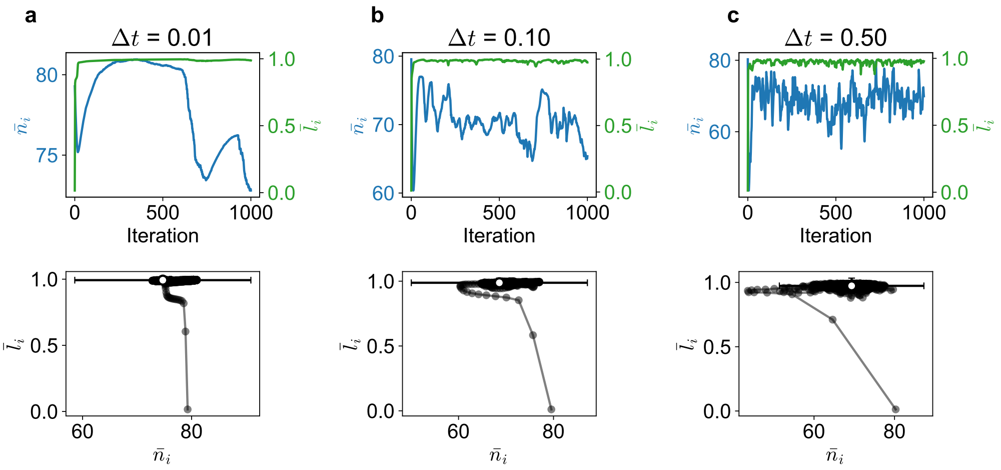

Figure S3. The trajectory of average number of neighbors $\bar{n}$ and average alignment $\bar{l}$ as function of iteration time for step-sizes (**a**) $\Delta t = 0.01$, (**b**) $\Delta t = 0.1$, and (**c**) $\Delta t = 0.5$ along with the corresponding phase-space plots (below) for $\alpha, \beta, \gamma, \delta = 0.5, 0.005, 0.25, 1.$.

1. Reynolds, C.W. Flocks, herds and schools: A distributed behavioral model. In Proceedings of the 14th annual Conference on Computer Graphics and Interactive Techniques, Anaheim, CA, USA, 27–31 July 1987; pp. 25–34.
2. Bialek, W.; Cavagna, A.; Giardina, I.; Mora, T.; Silvestri, E.; Viale, M.; Walczak, A.M. Statistical mechanics for natural flocks of birds. *Proc. Natl. Acad. Sci. USA* **2012**, *109*, 4786–4791.
